# The impact of smoking on annual healthcare cost: an econometric model analysis in China, 2015

**DOI:** 10.1186/s12913-021-06199-5

**Published:** 2021-02-28

**Authors:** Shiyao Huang, Han Wei, Tingting Yao, Zhengzhong Mao, Qun Sun, Lian Yang

**Affiliations:** 1 School of Management, Chengdu University of Traditional Chinese Medicine, Chengdu, People’s Republic of China; 2 Institute for Health and Aging, University of California, San Francisco, California USA; 3 Huaxi School of Public Health, Sichuan University, Chengdu, People’s Republic of China; 4 School of Public Health, Chengdu University of Traditional Chinese Medicine, No.1166, Liu Cheng Da Dao, Wenjiang District, Chengdu, People’s Republic of China

**Keywords:** Smoking, Medical expenditures, Econometric approach, China

## Abstract

**Background:**

Smoking exerts substantial medical burdens on society. Precise estimation of the smoking-attributable medical expenditures (SAME) helps to inform tobacco control policy makers. Based on the epidemiological approach, prior studies in China only focused on a few smoking-related diseases to estimate SAME. In contrast, this study used the econometric approach, which is capable of capturing all of the potential costs.

**Methods:**

Three waves of panel data from the 2011–2015 national China Health and Retirement Longitudinal Study (CHARLS) were used. A total of 34,503 observations aged 45 and above were identified. Estimates from econometric models were combined to predict the smoking-attributable fraction (SAF) and medical expenditures attributable to smoking by sex, registered residency and healthcare service categories. All monetary amounts were adjusted to 2015 dollars.

**Results:**

In 2015, the overall smoking-attributable fraction (SAF) of China was 10.97%, ranging from 5.77% for self-medication to 16.87% for inpatient visits. The smoking-attributable medical expenditure (SAME) was about $45.28 billion, accounting for 7.24% of the total health expenditure. The SAME was $226.77 per smoker aged 45 and above. The regression results suggest that being a former smoker has the greatest impact, which decreases over time after quitting however, on the value of medical expenditures.

**Conclusions:**

Smoking-attributable medical expenditures was substantial and placed a heavy burden on Chinese society. Comprehensive tobacco control policies and regulations are still needed to promote progress toward curbing the tobacco related losses.

## Background

China is the largest tobacco producer and consumer in the world. In 2015, it produced 2.6 trillion cigarettes (with 99% of which consumed domestically), which accounted for 46.32% of the world’s total cigarette production [1]. According to Chinese Center for Disease Control and Prevention (China CDC) report, the Chinses smoking prevalence of adults aged over 15 years is 26.6% in 2017 [2]. It is estimated that smoking causes almost 1 million deaths per year in China, and this number will continue to rise [3]. Because of the high prevalence and risk of smoking, China has always been the main battlefield of tobacco control for World Health Organization (WHO).

Due to its harmful health effects, smoking also incurs enormous medical costs, called smoking attributable medical expenditures (SAME). A precise SAME estimation is important for tobacco control policy makers. There are two common estimating methods: *the epidemiological approach* and *the econometric approach* [4]*.* Based on the smoking prevalence and relative risks (RR) of certain diseases, the epidemiological approach is used to measure the medical burden of smoking-related diseases, such as cancer, cardiovascular diseases, and respiratory diseases. However, besides the determined diseases, smoking causes or exacerbates health conditions through different pathways. It is difficult to capture all of the potential costs with this approach, which results in an underestimation of the medical burden caused by smoking. The econometric approach is conducted through econometric models regardless of the types of diseases caused by smoking. It is based on individual-level survey data, controlling for many covariates including sociodemographic characteristics as well as other risk behaviors. This approach allows a more robust health care cost estimation.

Four studies have estimated the SAME in China. With the epidemiological approach, Chen et al. [5] estimated SAME in 1988 to be $0.3 billion. From then on, researchers used the same approach to update the SAME in 1989, 2000, 2003, and 2008 to $ 0.8 billion, $ 1.7 billion, $ 4.2 billion, and $ 6.2 billion, respectively [6–8]. No study on SAME used the econometric approach in China. Moreover, China’s economy as well as the access to medical care have developed rapidly since 2008 [9]. It is necessary to monitor and update SAME. This paper aims to provide precise estimates for SAME in China in 2015 with the econometric approach by using data from the China Health and Retirement Longitudinal Study (CHARLS) conducted in 2011, 2013 and 2015. We estimated the medical expenditure of smoking from the healthcare perspective. The research results will help to increase the government’s impetus to curb the tobacco epidemic.

## Methods

### Overall

Panel data, also called longitudinal data in epidemiology, refers to data involving two dimensions that include time series and cross-section observation. It allows researchers to eliminate the impact from unobservable variables [10]. In this analysis, we value the impact of individual disease susceptibility on medical expenditure, but the degree is hard to quantify. Thus, panel data was adopted to reduce bias. Fixed-effects models, random-effect models and pooled ordinary least squares (OLS) are the common methods applied to panel data. Time-invariant variables do not affect the dependent variables and cannot be included in the fixed effects model. As smoking is addictive, smoking behaviors are difficult to change. Analyzing them with the select panel data fixed effect model will result in a loss of samples with unchanged smoking status and, hence, fitting model parameters that deviate from reality [11]. For panel data, pooled OLS models are generally considered to be biased in comparison to the two above mentioned models [10–12]. To ensure the robustness of the analysis results, this study used the random-effects model.

The smoking-attributable fraction (SAF) and SAME were assessed by type of health care services: inpatient hospitalizations, outpatient visits, and self-medications. The former two types generated prescribed medicine costs during health care utilization period. Self-medications refer to patients purchasing drugs on their own without seeing a doctor. Because the mean age for Chinese smokers to start smoking was around 20 [13], and the negative effects of smoking appear long after its onset, this study only focused on adults aged above 45.

All monetary amounts were adjusted to 2015 dollars using the medical care component of the Consumer Price Index (CPI) provided by the National Bureau of Statistics [14]. All data were analyzed using STATA (version 13.0, MP).

### Data

Most data were derived from CHALRS, which is a follow-up survey that collects data on adults aged 45 and above at multiple centers in China (in 28 provinces / municipalities / autonomous regions, covering 150 cities and 450 villages) [15]. The study ensured national representation with the method of multi-stage cluster sampling. Zhao et al. [16] have introduced their sampling methods and questionnaires in detail. CHARLS was activated in 2011 and tracks the subjects in every 2 years. So far, three waves of survey data have been publicly released covering the data in 2011, 2013 and 2015. After the exclusion of 1638 non-cigarette smokers, calculation results showed that 71.57% of the respondents had successfully been followed up in the three waves of surveys. We eventually established a balanced panel with the inclusion of 11,501 individuals (34,503 observations over 3 years). The survey used computer-assisted person interview (CAPI) to collect personal information, including demographic background, smoking behavior, health status, and utilization of medical services. The population data were derived from China Statistical Yearbooks and China Demographic and Employment Statistical Yearbooks [14, 17, 18].

### Measurement

#### Smoking status

Smoker was defined as “a person who smoked 100 cigarettes in their lifetime”. Based on the smoking information, CHARLS respondents were divided into 6 categories: never smokers; current light smokers whose smoking index were lower than 200; current heavy smokers whose smoking index were higher than 200; former smokers who quitted within 5 years; and former smokers who quitted more than5 years ago. Smoking index is obtained by multiplying the number of cigarettes smoked per day by years of smoking.

#### Independent variables

Sociodemographic characteristics included sex (female, male), age (45 ~ 54 years old, 55 ~ 64 years old, ≥65 years old), registered residents (rural, urban), education level (primary school and below; middle school and above), marital status (single, separated, divorced, widowed; married or cohabitating), poverty status (no, yes), health insurance coverage status (no, yes), and economic region (west, middle, east). Four indicators were chosen to reflect poverty [19]: whether the main source of cooking fuel is traditional energy such as crop residue or wood burning; whether the toilet is not flushable; whether the residence has no running water; whether the respondent owns less than two asset in the following list: computer, refrigerator, washing machine, TV, air conditioner, mobile phone, music instrument, video camera, stereo system. If respondent answered “yes” to any two of those four questions, he or she was in poverty status. Other risk factors included body mass index (BMI) status (normal weight, underweight, overweight), and drinking status (drinker, never drinker). BMI means weight (kg) / height(m)^2^. The normal BMI of Chinese people ranges from 18.5 to 23.9 kg/m^2^ [20]. The drinker was defined as one who drinks any alcoholic beverage (such as beer, wine, or liquor) more than once a month.

### Estimation for smoking-attributable fraction

Random-effects models were used for the analysis:

1 $$ {\mathrm{DExp}}_{\mathrm{it}}={\gamma}_0+{\gamma}_1{Smoking}_{it}+{\gamma}_2{X}_{it}+{\gamma}_3{Y}_{it}+{\gamma}_4{Int}_{it}+{\alpha}_i+{u}_{it} $$

2 $$ \ln {\mathrm{Exp}}_{\mathrm{i}\mathrm{t}}={\upbeta}_0+{\upbeta}_1{\mathrm{Smoking}}_{\mathrm{i}\mathrm{t}}+{\upbeta}_2{\mathrm{X}}_{\mathrm{i}\mathrm{t}}+{\upbeta}_3{\mathrm{Y}}_{\mathrm{i}\mathrm{t}}+{\upbeta}_4{\mathrm{Int}}_{\mathrm{i}\mathrm{t}}+\upalpha {\prime}_{\mathrm{i}}+\mathrm{u}{\prime}_{\mathrm{i}\mathrm{t}} $$

Where DExp_it_ is a dummy variable, indicating whether person *i* used the health care service at year t (an indicator of positive healthcare spending). Exp_it_ is the medical expenditures if the person used the service. Smoking denotes smoking status that is assumed to be exogenous. X is a matrix of sociodemographic characteristics variables. Y is a matrix of risk factors. Int is the interaction between smoking status and age. We have introduced the interaction of smoking and age in the model because physical functions gradually decline with age. Among age groups, the association between smoking and the probability of medical visits and medical expenses is different. If the interaction is statistically significant, we will further develop a stratified analysis by age. Otherwise, we will not deal with it. *α*_*i*_ is the random heterogeneity specific to the *i*th individual and is time-invariant. *u*_it_ is the error term. Eq. (1) was estimated by logit model with random effects, and Eq. (2) was estimated by generalized least square (GLS) model with random effect. The codes in STATA (version 13.0, MP) are “xtlogit y x, re” and “xtreg y x, re r”, respectively.

Estimated parameters from two equations were combined to predict two sets of individual medical expenditures [21]. The first one is the estimated “factual” costs of current and former smokers. Meanwhile, the “hypothetical” costs were obtained by setting the values of all smokers as 0 in mathematical form, which means assuming them to be never smokers. SAF is the ratio of the difference between “factual” costs and “hypothetical” costs to the “factual” costs.

### Estimation for smoking-attributable medical expenditures

The SAME for each subgroup stratified by health care service types was estimated by multiplying the SAF by the corresponding total medical expenditures (THE) according to the following formula [7, 8]:

3 $$ {\displaystyle \begin{array}{c}\mathrm{SAME}=\sum \mathrm{THE}\times \mathrm{SAF}\\ {}=\mathrm{PV}\times \mathrm{QV}\times 12\times \mathrm{POP}\times {\mathrm{SAF}}_v+\mathrm{PH}\times \mathrm{QH}\times \mathrm{POP}\times {\mathrm{SAF}}_h+\mathrm{PM}\times \mathrm{QM}\times 12\times \mathrm{POP}\times {\mathrm{SAF}}_m\end{array}} $$

Where PV is the average expenditures per outpatient visit; QV stands for the average number of outpatient visits per person in 1 month; PH represents the average expenditures per inpatient hospitalization; QH is the average number of inpatient visits per person in 12 months; PM is the average medication expenditure per person with positive self-medication expenditures in 1 month; QM is the proportion of persons with positive self-medication expenditures in 1 month; POP is the population aged 45 years and above in 2015; v indicates outpatient visits; h stands for inpatient visits; m indicates self-medication.

In CHARLS, the medical expenditures of all health care services were reported by respondents, and consisted both insurance payment and out-of-pocket payment of individual patients. To reflect actual expenditures with self-reported medical expenditure data, we conducted an adjustment process. The main idea is to calculate the adjusted factor by dividing the estimated national expenditure by the national healthcare expenditures of people aged 45 and above and then apply it to the estimated average expenditures from the CHALRS data. The numerator was derived by removing the SAF in Eq. (3), while the denominator was calculated by multiplying the official figure of all-aged national healthcare expenditures and 59.99%. The figure 59.99% comes from the accounting results of current Chinese health expenditure in 2012 based on System of Health Accounts 2011 (SHA 2011) [22]. Assuming the ratio was relatively stable in the duration of a few years, we applied it to the data for 2015. The adjusted factor is 1.23 in 2015.

## Results

Table 1 reports the characteristics of respondents from the CHALRS by year. In the baseline year, among the respondents, 28.47% were current smokers (4.10% for light smokers and 24.37% for heavy smokers), 6.84% were former smokers (3.90% quit≤5 years and 2.94% quit> 5 years), and 64.40% were never smokers. The research results on proportion after weight-adjustment show that, over time, the proportions of non-smokers and heavy smokers have decreased whereas the proportions of mild smokers and quitters have increased. The distribution characteristics of the remaining variables remained stable in different years. A higher proportion of residents were married nonalcoholic women in rural areas with lower education levels, who were not suffering from poverty and had medical insurance coverage. There is a relatively even distribution of residents among age groups and economic regions.

**Table 1:** Weighted Descriptive Statistics of Study Sample, CHARLS, 2011, 2013 and 2015 ^a^

Type of variable	Level of variable	2011	2013	2015	
Smoking status	Never smokers	64.4	62.06	60.18	
	Current light smokers	4.10	4.93	5.03	
	Current heavy smokers	24.37	23.12	21.44	
	Former smokers, quit≥5 yr	3.90	4.28	6.37	
	Former smokers, quit< 5 yr	2.94	5.62	6.98	
Sex	Females	55.44	55.44	55.44	
	males	44.56	44.56	44.56	
Age group	45 ~ 54 yrs	39.34	31.85	26.29	
	55 ~ 64 yrs	37.50	39.07	37.69	
	65+ yrs	23.15	29.08	36.02	
Registered residents	Rural	57.52	57.52	57.52	
	Urban	42.48	42.48	42.48	
Education level	Primary school and below	65.12	64.79	63.40	
	Middle school and above	34.88	35.21	36.60	
Marital status	Single/separated/divorced/widowed	10.70	11.79	13.22	
	Married/cohabitating	89.30	88.21	86.78	
Living poverty status	No	77.65	85.51	88.61	
	Yes	22.35	14.49	11.39	
Having health insurance	No	5.80	3.31	7.73	
	Yes	94.20	96.69	92.27	
Economic regions (ER)	West ER	29.55	29.55	29.55	
	Middle ER	27.86	27.86	27.86	
	East ER	42.59	42.59	42.59	
BMI status	Normal weigt	49.40	46.73	46.80	
	Underweight	6.11	5.55	6.38	
	Overweight	44.49	47.72	46.82	
Drinking status	Never drinker	75.66	74.58	74.89	
	Drinker	24.34	25.42	25.11	

^a^ Estimates are weighted using individual sampling weights with household and individual response adjusted. The sampling weights were provided by CHALRS. More details could be found in the CHALRS User’s Guidance

Table 2 provides the estimated parameters and test statistics for each of the six models. Adjusted odd ratio (aOR) from logit model was used to measure the association between exposures and outcomes. The GLS regression coefficient obtained is “semi-elastic” coefficient, which indicates the percentage of increase or decrease in medical expenses when the individual is a smoker. Compared with never smokers, current smokers exhibit no difference in medical visit probability and medical expenses in almost all types of medical services (aOR is approximately equal to 1, GLS β is approximately equal to 0). Being a former smoker has a positive effect in all equations, and most of the estimated coefficients were statistically significant. The estimated coefficient of this covariate in determining the level of hospitalization is also the highest among all smoking variable coefficients. Table 2 also shows that factors such as age, urban residence, married or cohabiting, better educational background and owning medical insurance had positive effects on healthcare visits and medical expenses. Meanwhile, factors such as male sex, economically developed regions, BMI in normal status, and drinker, however, exerted a significant negative impact on the healthcare visits and medical expenses. Almost all of the interactions are not significant in models.

**Table 2:** Parameter estimates and test statistics, CHARLS, 2011, 2013 and 2015 ^a^

Covariables	Outpatient visits	Log of outpatient expenditures	Inpatient hospitalizations	Log of inpatient expenditures	self-medication	Log of self-medication expense
	Logit aOR [*P* Value]	GLS β [*P* Value]	Logit aOR [*P* Value]	GLS β [P Value]	Logit aOR [*P* Value]	GLS β [*P* Value]
**Smoking status**						
Never smokers	Reference group					
Current light smokers	0.907	−0.0298	1.082	−0.0593	1.028	−0.0250
Current heavy smokers	0.740* ^b^	0.103	0.875	0.0579	1.097	−0.181*
Former smokers, quit< 5 yr	1.421*	0.886*	1.728*	0.445*	1.287	0.206*
Former smokers, quit≥5 yr	1.574*	0.137	1.138	0.518*	1.582*	0.0456
**Sex**						
Females	Reference group					
Males	0.742*	−0.0903	0.975	0.157*	0.801*	−0.0818*
**Age group**						
45 ~ 54 yrs	Reference group					
55 ~ 64 yrs	0.998	0.159*	1.395*	0.0952	1.255*	0.156*
65+ yrs	1.106*	0.352*	1.940*	0.0862	1.238*	0.270*
**Registered residents**						
Rural	Reference group					
Urban	1.015	0.148*	1.122*	0.283*	1.160*	0.135*
**Education level**						
Primary school and below	Reference group					
Middle school and above	0.883*	0.222*	0.963	0.146*	1.099*	0.125*
**Marital status**						
Single/Separated/divorced/widowed	Reference group					
Married/cohabitating	0.984	0.154*	0.883	0.143*	1.075	0.117*
**Living poverty status**						
No	Reference group					
Yes	1.035	−0.0394	1.000	− 0.127*	1.140*	0.0162
**Having health insurance**						
No	Reference group					
Yes	1.322*	−0.0147	1.595*	0.0573	1.408*	0.0464
**Economic regions (ER)**						
West ER	Reference group					
Middle ER	0.672*	−0.118*	0.712*	0.0241	0.598*	−0.225*
East ER	0.480*	0.0495	0.530*	0.360*	0.595*	−0.274*
**BMI status**						
Normal weigt	Reference group					
Underweight	1.125	0.172*	1.217*	0.0970	0.903	0.0881
Overweight	0.979	0.122*	1.202*	0.133*	1.305*	0.0771*
**Drinking status**						
Never drinker	Reference group					
Drinker	0.876*	−0.305*	0.649*	−0.156*	0.953	−0.243*
**Interaction term (IT)** ^**a**^						
IT 1	1.053	0.335	1.194	0.0171	1.058	− 0.0320
IT 2	1.024	− 0.0807	0.991	− 0.126	1.055	−0.130
IT 3	1.200	−0.173	0.898	−0.178	0.973	0.0992
IT 4	1.206	−0.148	1.019	−0.0289	1.105	0.136
IT 5	1.043	−0.190	1.348	0.0139	0.989	0.102
IT 6	0.83	−0.405	1.381	0.0948	0.835	0.113
IT 7	0.798	0.217	1.452	−0.543*	0.945	0.162
IT 8	1.093	0.190	1.728*	−0.223	1.039	0.322*
**Constant**	**0.433**	5.072*	0.0738*	7.887*	0.624*	4.057*

^a^ IT 1 denotes the interaction between current light smokers and the 55 ~ 64 year age group; IT 2 denotes the interaction between current light smokers and the 65+ year age group; IT 3 denotes the interaction between current heavy smokers and the 55 ~ 64 year age group; IT 4 denotes the interaction between current heavy smokers and the 64+ year age group; IT 5 denotes the interaction between former smokers who quitted within 5 yr and 55 ~ 64 year age group; IT 6 denotes the interaction between former smokers who quitted within5yrs and the 64+ year age group; IT 7 denotes the interaction between former smokers who quitted more than 5 years ago and the 55 ~ 64 year age group; IT 8 denotes the interaction between former smokers who quitted more than 5 years ago and the 64+ year age group. ^b^ t-Statistics in parentheses: *p < 0.05

We calculated the SAF and SAME by type of health care services. Nationally, Table 3 shows that the SAF was 10.97%, and the highest SAF among the types of healthcare services was seen in inpatient (16.87%), while the lowest SAF was seen in self-medication (5.77%). Accordingly, SAME amounted to $45.28 billion and $226.77 per smoker. The outpatient SAME is the highest (23.15 billion) due to the large visit amount, followed by inpatients and self-medication SAME, which amounted to 21.52 billion and 0.61 billion, respectively. After the number of smoker was adjusted with different types of health care service, the SAME is highest in inpatient ($568.82) per smoker, followed by outpatients ($376.15) and self-medication ($6.05). This is consistent to the price distribution of Chinese medical services.

**Table 3:** SAF, SAME and per capital SAME, by type of healthcare service, 2015

Smoking status	SAF (%)	SAME (billion $)	Per Smoker ($)
Outpatient	9.07	23.15	376.15
Inpatient	16.87	21.52	568.82
Self-medication	5.77	0.61	6.05
Overall	10.97	45.28	226.77

Table 4 shows the SAF and SAME by sex, urban/rural district and healthcare services. Among all types of healthcare services, the SAF of men is about ten times that of women. The SAF of residents who lived in urban areas is almost equal to that of residents who lived in rural. Accordingly, our calculation results show that the overall SAME for males is $41.48 billion, which is 13 times that of females ($4.88 billion), and that the overall SAME for urban ($24.31 billion) and rural residents ($22.23 billion) are almost equivalent. The distribution of outpatient, inpatient, or self-medication SAME is consistent with that of overall SAME. From the perspective of populations, SAF is the highest in inpatient hospitalization, followed by outpatient visit and self-medication. SAME ranks first in outpatient visit, which is higher than inpatient hospitalization, and self- medication.

**Table 4:** SAF and SAME for CHARLS participants by sex, registered regions and health care types, 2015

	SAF (%)			SAME ($ billions)			
	Outpatient	Inpatient	Self-medication	Outpatient	Inpatient	Self-medication	Total ^a^
Males	20.02	29.33	12.44	21.99	18.94	0.56	41.48
Females	1.85	3.27	1.04	2.69	2.12	0.07	4.88
Total	–	–	–	24.67	21.06	0.63	46.36
Urban	9.89	17.51	6.14	11.23	12.73	0.35	24.31
Rural	8.53	16.32	5.50	12.14	9.78	0.31	22.23
Total	–	–	–	23.37	22.51	0.66	46.54

^a^ The sum of individual categories may not equal the total due to rounding

## Discussions

This study provides the first SAF and SAME estimates based on econometric approach in China. From the healthcare perspective, the overall SAF of medical expenditures in China was estimated to be 10.97%. The results are similar to research results (ranging from 6.5 to 11.8%) conducted in the United States or Canada that also used the econometric approach [4, 21, 23, 24]. Accordingly, the annual medical expenditures for China in 2015 attributed to smoking was estimated to be $45.28 billion, which means an averaging of $226.77 per smoker aged 45 and above. The total value of SAME accounted for 7.24% of total medical expenditures in the same period, which is higher than the results in previous Chinese studies — 3.1 and 3.0% in 2000 and 2008, respectively [7, 8].

Firstly, the difference in the cost estimation approach caused this discrepancy. Prior studies used *the epidemiological approach*, and cost estimates were limited to malignant tumors, circulatory diseases, and respiratory diseases associated with smoking. However, smoking can also cause gastroesophageal reflux disease, periodontal disease, mental illness, reproduction and erectile dysfunction [25–27]. Hence, prior analyses may have underestimated the medical costs. Moreover, social factors may also have led to higher economic costs of diseases in this study. Since 2009, owing to the benefits of New Medical and Health System Reform policy [9], the number of visits in medical and health centers in China has increased from 3.5 billion visits per year for outpatient and 0.1 billion per year for inpatient in 2008 to 7.7 billion visits per year for outpatient and 0.2 billion per year for inpatient in 2015 [17, 28]. Meanwhile, the rapid development of medical technology has led to increased costs of medical services. The average outpatient cost per visit and the average inpatient cost per capita in general hospitals have increased by 62.11 and 63.87% respectively, which led to a higher estimation of direct medical costs attributable to smoking [17, 28].

We have measured SAF and SAME among different types of medical services, sex groups, and different urban and rural areas. For different types of medical services, SAF ranges from 5.77 to 16.87%, and SAME ranges from $0.61 billion to $23.15 billion. The distribution is the same as that in prior researches. SAF and SAME are much higher in men than in women, mainly because the smoking rate of men (52.1%) is greater than that of women (2.7%), indicating that men are still the main population on which tobacco control strategies need to be implemented. Based on the calculations in this study, there are only small gaps between SAF and SAME in urban and rural areas respectively, which is consistent with the results in previous studies on the economic burdens attributable to smoking inflicted diseases in China [7, 8].

As we all know, smoking is an addictive behavior, which has a long-term impact on the health system. As is suggested in our regression results, when compared with non-smokers, light current smokers show no difference in terms of healthcare utilization and medical expenditure. This is possible because the effect of smoking has not yet occurred among light current smokers in their short smoking history; the heavy current smokers have a lower probability of healthcare utilization but spend more on medical services. According to theories in Behavioral Economics, smokers are prone to psychological cognitive passivation on the health damage caused by smoking. They are likely to have a blind faith in their health, so they are more reluctant to see a doctor [29]. Nevertheless, the higher medical expenditures among heavy current smokers indicate that heavy smoking actually causes smokers to have more serious diseases, or the diseases may have developed into chronic diseases that are difficult to cure, such as COPD and cancer. These diseases require investment in treatment after smoke cessation, which means that the healthcare system needs to cover medical treatment for many years (from the beginning of smoking, to the occurrence of diseases, and even years after smoking cessation). The GLS model also suggested that the impact of former smokers on the cost of medical expenditures decreases over time after smoking cessation. Adhering to smoking cessation is helpful for rejuvenating physical health [30]. However, as smoking cessation is a complicated process, many smokers have difficulties persisting without internal trigger or external pressures [31]. Researches have shown that there is still a high smoking relapse rate of 25.6% in China [32]. The questions of how to increase the determination of smokers to quit smoking and reduce smoking relapse rate are also directions of intervention that deserve further attention.

This paper has a panel data with individual information on the risk factors (tobacco consumption, specific data, intensity, and accumulated) and the covariates, which allows a more robust health care cost estimation through econometric models. Nonetheless, this paper has several limitations. First, we have utilized a nationwide follow-up data, the biggest disadvantage of which is loss to follow-up. This study retained the survey data of respondents who had been followed up for 3 years, with a follow-up rate of 71.56%, which is already a very high rate in large-scale social science researches. In epidemiological studies, however, we would expect a loss to follow up rate of no more than 20%. Secondly, most studies considered the population aged 35 years and over, but this study only focused on adults aged 45 years and over. It could not be generalized to the whole population. Thirdly, tobacco use is a kind of mental disease because nicotine induces addiction. China has set up smoking cessation outpatient clinic since 1996 but, however, smokers were hardly willing to visit it. Our results did not fully include this part. In addition, we did not estimate the effect of secondhand smoking on exposed women and children either. All the factors would cause underestimation to the total cost.

Smoking rate reduction, which lowers not only health hazards but also economic costs, has become one of the strategic goals in the Healthy China 2030 plan [33]. Studies showed that increasing the tobacco tax prevalence is likely to be the most effective way to reduce tobacco use [34, 35]. In 2015, China’s second increase in tobacco tax caused a slight increase in tobacco prices, which reduced tobacco sales in the short term [36]. However, it rebounded later [37]. This fluctuation indicates that tax increase was helpful to China’s tobacco control, but the increase in household income exceeded the rise of cigarette prices. In other words, in comparison to resident purchasing power, the relative consumer price of cigarettes decreased [38]. China is currently implementing a 56 and 36% tax prevalence on Class A and B cigarettes respectively, but there is still much room for improvement, based on the 70% retail price tax prevalence proposed by the World Health Organization [39]. Therefore, it is recommended that the government further increase the tobacco tax and keep tracking changes in residents’ ability to pay for cigarettes by adopting a dynamic strategy in the adjustment of tobacco consumption tax rate. Meanwhile, the introduction of a tobacco tax as a “special tax” can be considered. The “special tax” is used to support smoking cessation activities, health promotion and disease control, and the treatment of smoking-related diseases.

In addition, it is also recommended to coordinate communities and medical staff to make more efforts to persuade smokers, especially current smokers with high daily tobacco consumption and long smoking history, to quit smoking as soon as possible. Setting up more smoking cessation clinics and encouraging those who have quitted smoking to carry on will help to avoid greater losses.

## Conclusions

Our study shows that the SAME is substantial, resulting in negative impacts on individuals and the healthcare system. Efforts to reduce smoking prevalence are warranted. It is mainly recommended to further raise tobacco taxes or set up a “special tax” as financial supports for tobacco control. Moreover, econometric approach has the advantage of providing more robust and comprehensive cost estimates. We hope high-quality, large-scale, follow-up research activities covering smoking information to continue in China and the use of the econometric approach in the SAME estimation can be routinized.

## Data Availability

The data that support the findings of this study are available at http://charls.pku.edu.cn/index/en.html. Restrictions apply to the availability of the data. The data used is, however, available from the authors upon reasonable request and with permission of National School of Developments, Peking University.

## Abbreviations

aOR: Adjusted Odd Ratio
BMI: Body Mass Index
CAPI: Computer-assisted Person Interview
CHARLS: China Health and Retirement Longitudinal Study
CPI: Consumer Price Index
GLS: Generalized Least Square
RR: Relative Risk
OLS: Ordinary Least Squares
SAME: Smoking-attributable Medical Expenditures
SAF: Smoking-attributable Fraction
SHA 2011: System of Health Accounts 2011
THE: Total Health Expenditures
WHO: World Health Organization
